# Development of a new multiplex quantitative PCR for the detection of *Glaesserella parasuis*, *Mycoplasma hyorhinis*, and *Mycoplasma hyosynoviae*

**DOI:** 10.1002/mbo3.1353

**Published:** 2023-05-15

**Authors:** Simone Scherrer, Sarah Schmitt, Fenja Rademacher, Peter Kuhnert, Giovanni Ghielmetti, Sophie Peterhans, Roger Stephan

**Affiliations:** ^1^ Section of Veterinary Bacteriology, Institute for Food Safety and Hygiene, Vetsuisse Faculty University of Zurich Zurich Switzerland; ^2^ Institute of Veterinary Bacteriology, Vetsuisse Faculty University of Bern Bern Switzerland; ^3^ Institute for Food Safety and Hygiene, Vetsuisse Faculty University of Zurich Zurich Switzerland

**Keywords:** *Glaesserella parasuis*, multiplex qPCR, *Mycoplasma hyorhinis*, *Mycoplasma hyosynoviae*, *vtaA*

## Abstract

*Glaesserella parasuis*, *Mycoplasma hyorhinis*, and *Mycoplasma hyosynoviae* are important porcine pathogens responsible for polyserositis, polyarthritis, meningitis, pneumonia, and septicemia causing significant economic losses in the swine industry. A new multiplex quantitative polymerase chain reaction (qPCR) was designed on one hand for the detection of *G. parasuis* and the virulence marker *vtaA* to distinguish between highly virulent and non‐virulent strains. On the other hand, fluorescent probes were established for the detection and identification of both *M. hyorhinis* and *M. hyosynoviae* targeting 16S ribosomal RNA genes. The development of the qPCR was based on reference strains of 15 known serovars of *G. parasuis*, as well as on the type strains *M. hyorhinis* ATCC 17981^T^ and *M. hyosynoviae* NCTC 10167^T^. The new qPCR was further evaluated using 21 *G. parasuis*, 26 *M. hyorhinis*, and 3 *M. hyosynoviae* field isolates. Moreover, a pilot study including different clinical specimens of 42 diseased pigs was performed. The specificity of the assay was 100% without cross‐reactivity or detection of other bacterial swine pathogens. The sensitivity of the new qPCR was demonstrated to be between 11–180 genome equivalents (GE) of DNA for *M. hyosynoviae* and *M. hyorhinis*, and 140–1200 GE for *G. parasuis* and *vtaA*. The cut‐off threshold cycle was found to be at 35. The developed sensitive and specific qPCR assay has the potential to become a useful molecular tool, which could be implemented in veterinary diagnostic laboratories for the detection and identification of *G. parasuis*, its virulence marker *vtaA*, *M. hyorhinis*, and *M. hyosynoviae*.

## Graphical abstract

The aim of the study was to develop a multiplex PCR for identification of *Glaesserella parasuis* and its virulence marker *vtaA* distinguishing between highly virulent and non‐virulent strains. Furthermore, *Mycoplasma hyorhinis* and *Mycoplasma hyosynoviae* can be identified targeting 16S rRNA. The developed multiplex PCR provides a simple one‐tube assay, which can be performed rapidly and cost‐efficiently allowing an efficient throughput of samples in veterinary diagnostic laboratories.

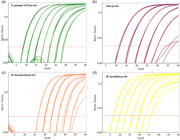

## 1 INTRODUCTION

*Glaesserella parasuis* is the etiologic agent of Glässer's disease in piglets causing significant economic losses to the swine industry. Infected pigs can develop polyserositis, polyarthritis, meningitis, pneumonia, or septicemia. *G. parasuis* comprises strains with heterogeneous virulence capacities ranging from non‐virulent to highly virulent (Galofré‐Milà et al., 2017). In 2015, a multiplex polymerase chain reaction (PCR) was established enabling the identification and serotyping of *G. parasuis* (Howell et al., 2015). Phenotypically indistinguishable isolates may be the cause of invasive systemic disease or be part of the colonizing flora of the upper respiratory tract of healthy pigs. Therefore, it is important to distinguish between virulence‐associated and non‐virulent commensal *G. parasuis* strains. Several *G. parasuis* virulence markers have been proposed, such as a putative hemolysin gene operon *hhdBA* (Sack & Baltes, 2009), lipopolysaccharide sialyltransferase gene *lsgB* (Martínez‐Moliner et al., 2012), a putative *espP2* gene coding for a putative extracellular serine protease‐like protein (Zhang et al., 2012) and a truncated outer membrane ferric hydroxamate receptor gene *fhuA* (Zhou et al., 2010), however, there is still little experimental data to find a correlation between clinical disease and these virulence genes. Furthermore, the so‐called leader sequence (LS)‐PCR was developed targeting two different sequences of the virulence‐associated trimeric autotransporters (*vtaA*) genes, allowing for the differentiation between putative virulent and non‐virulent *G. parasuis* strains (Galofré‐Milà et al., 2017). In the case of most well‐characterized group 1 *vtaA* genes, affirmation for their role in virulence was found (Galofré‐Milà et al., 2017; Olvera et al., 2012). The LS‐PCR was confirmed to be useful for virulence prediction also for global *G. parasuis* isolates (Macedo et al., 2021; Schuwerk et al., 2020). Alternatively, a high‐resolution melting assay was developed recently, aiming at serotyping *G. parasuis* and at the same time targeting *vtaA* for the prediction of potentially virulent strains (Scherrer et al., 2022).

*Mycoplasma hyorhinis* is a colonizer of the upper respiratory tract of pigs, which is found especially in the nasal cavity and tonsils (Pieters & Maes, 2019). Even though *M. hyorhinis* has long been known as a typical commensal of pigs, it is nowadays recognized as an emerging pathogen in intensive swine production (Dos Santos et al., 2015). *M. hyorhinis* is implicated in polyarthritis and polyserositis in recently weaned pigs, whereas in older pigs disease is usually characterized by mild arthritis alone (Pieters & Maes, 2019). Additionally, *M. hyorhinis* is frequently found in the lungs as a secondary pathogen (Kobayashi et al., 1996). Similar to *M. hyorhinis*, *Mycoplasma hyosynoviae* is often found in the nasal cavity, tonsils, and conducting airways of colonized pigs. *M. hyosynoviae* is generally recovered in joints of finishing pigs over 10 weeks of age causing nonpurulent arthritis. *M. hyosynoviae* is ubiquitously distributed among the pig population (Pieters & Maes, 2019). Arthritis triggered by *M. hyosynoviae* is frequently associated with stress factors such as an increased density of pigs in a stock, mixing herds with nonlitter mates, or temperature changes (Ross, 1973). Several PCR tests have been developed for the detection of *M. hyorhinis* and *M. hyosynoviae* in different sample materials including joint fluid, nasal‐ and tonsillar swabs, or oral fluids associated with lameness and arthritis. However, when using oral fluid samples in a PCR, only limited accuracy on different age groups could be achieved (Gomes Neto et al., 2015; Pillman et al., 2019).

Due to the fastidious character of these three pathogens, the cultivation is difficult, time‐critical, and has a low sensitivity. Therefore, detection using PCR, ideally also distinguishing between highly virulent and non‐virulent *G. parasuis* isolates, would be an attractive alternative for their diagnosis.

In 2019, the Swiss Federal Food Safety and Veterinary Office started a pilot project to increase the necropsy rates of diseased swine to better assess the health situation in the pig population and increase awareness for specific diseases. Once the project started, it became evident that some routine diagnostic tests for important swine pathogens such as *G. parasuis*, *M. hyorhinis*, and *M. hyosynoviae* were not available. In the course of a pilot study, various clinical specimens of 42 diseased pigs were analyzed in detail. Our aim was therefore to optimize and implement the diagnosis of these three relevant swine pathogens.

## 2 MATERIALS AND METHODS

### 2.1 Strains and field isolates

Seventeen reference strains including *G. parasuis* serovars 1–15, *M. hyorhinis* ATCC 17981^T^, and *M. hyosynoviae* NCTC 10167^T^ were used for developing a multiplex quantitative PCR (qPCR) system (Table 1). For further evaluation of the developed qPCR, 21 *G. parasuis* field isolates obtained from routine diagnostic submissions to the Section of Veterinary Bacteriology, University of Zurich, between 2007 and 2022, three field isolates of *M. hyosynoviae* and another 26 field isolates of *M. hyorhinis*, covering a broad range of genotypes (Trüeb et al., 2016), were used (Table 2).

**Table 1:** Reference strains used for the development of the multiplex qPCR assay.

Species	Strain	Serovar	LS‐PCR^a^
*Glaesserella parasuis*	nr. 4	1	V
*Glaesserella parasuis*	SW140	2	V
*Glaesserella parasuis*	SW114	3	NV
*Glaesserella parasuis*	SW124	4	V
*Glaesserella parasuis*	Nagasaki	5	V
*Glaesserella parasuis*	131	6	NV
*Glaesserella parasuis*	174	7	V
*Glaesserella parasuis*	C5	8	NV
*Glaesserella parasuis*	D74	9	NV
*Glaesserella parasuis*	H555	10	NV
*Glaesserella parasuis*	H465	11	V
*Glaesserella parasuis*	H425	12	V
*Glaesserella parasuis*	84‐17975	13	V
*Glaesserella parasuis*	84‐22113	14	V
*Glaesserella parasuis*	84‐15995	15	V
*Mycoplasma hyorhinis*	ATCC 17981^T^	–	–
*Mycoplasma hyosynoviae*	NCTC 10167^T^	–	–

Abbreviations: LS‐PCR, leader sequence PCR; NV, non‐virulent; qPCR, quantitative PCR; V, virulent.

^a^ Virulence determined by LS‐PCR (Galofré‐Milà et al., 2017).

**Table 2:** Field isolates of *Glaesserella parasuis*, *Mycoplasma hyorhinis*, and *Mycoplasma hyosynoviae*.

Species	ID	Year	Origin	Sequence type	Serovar^a^	Virulence^b^
*Glaesserella parasuis*	SS626 SK1	2007	Lung	–	1	Virulent
*Glaesserella parasuis*	SS626 SK2	2007	Lung	–	7	Virulent
*Glaesserella parasuis*	PP396	2016	Joint	–	2	Virulent
*Glaesserella parasuis*	SS3873	2017	Joint	–	2	Virulent
*Glaesserella parasuis*	SS3875	2017	Joint	–	2	Virulent
*Glaesserella parasuis*	SS3939	2017	Joint	–	2	Virulent
*Glaesserella parasuis*	PP733	2018	Brain	–	2	Virulent
*Glaesserella parasuis*	PP749	2018	Lung	–	2	Virulent
*Glaesserella parasuis*	PP797	2019	Brain	–	13	Virulent
*Glaesserella parasuis*	PP808	2019	Lung	–	4	Virulent
*Glaesserella parasuis*	PP849	2020	Lung	–	7	Virulent
*Glaesserella parasuis*	SS5061	2020	Joint	–	13	Virulent
*Glaesserella parasuis*	PP879	2021	Lung	–	14	Virulent
*Glaesserella parasuis*	21‐640/1	2021	Lung	–	4	Virulent
*Glaesserella parasuis*	SS5603	2021	Joint	–	4	Virulent
*Glaesserella parasuis*	PP903	2021	Brain	–	7	Virulent
*Glaesserella parasuis*	22‐180	2022	Brain	–	7	Virulent
*Glaesserella parasuis*	22‐676/4	2022	Brain	–	2	Virulent
*Glaesserella parasuis*	22‐804	2022	Nasal	–	13	Virulent
*Glaesserella parasuis*	22‐1006	2022	Brain	–	4	Virulent
*Glaesserella parasuis*	22‐1176	2022	Nasal	–	7	Virulent
*Mycoplasma hyorhinis*	1165S18	2018	Serosa	98	–	–
*Mycoplasma hyorhinis*	893S18	2018	Pericard	89	–	–
*Mycoplasma hyorhinis*	4236J19	2019	Joint liquid	92	–	–
*Mycoplasma hyorhinis*	2625	2014	Lung	52	–	–
*Mycoplasma hyorhinis*	2783	2014	Lung	53	–	–
*Mycoplasma hyorhinis*	10003‐41	2014	BALF	54	–	–
*Mycoplasma hyorhinis*	10025‐1	2014	BALF	13	–	–
*Mycoplasma hyorhinis*	CH‐01‐05	2015	Lung	30	–	–
*Mycoplasma hyorhinis*	CH‐08‐13	2015	Lung	33	–	–
*Mycoplasma hyorhinis*	CH‐09‐01	2015	Lung	34	–	–
*Mycoplasma hyorhinis*	DE‐02‐05	2014	Lung	35	–	–
*Mycoplasma hyorhinis*	DE‐03‐06	2014	Lung	36	–	–
*Mycoplasma hyorhinis*	DE‐03‐08	2014	Lung	37	–	–
*Mycoplasma hyorhinis*	DE‐04‐16	2014	Lung	38	–	–
*Mycoplasma hyorhinis*	DE‐04‐31	2014	Lung	39	–	–
*Mycoplasma hyorhinis*	DE‐05‐48	2014	Lung	40	–	–
*Mycoplasma hyorhinis*	DE‐07‐13	2014	Lung	41	–	–
*Mycoplasma hyorhinis*	DE‐10‐05	2014	Lung	42	–	–
*Mycoplasma hyorhinis*	DE‐13‐30	2014	Lung	43	–	–
*Mycoplasma hyorhinis*	DE‐14‐31	2014	Lung	44	–	–
*Mycoplasma hyorhinis*	DE‐15‐12	2014	Lung	45	–	–
*Mycoplasma hyorhinis*	DE‐16‐34	2015	Lung	46	–	–
*Mycoplasma hyorhinis*	DE‐16‐40	2015	Lung	47	–	–
*Mycoplasma hyorhinis*	DE‐17‐13	2015	Lung	48	–	–
*Mycoplasma hyorhinis*	DE‐18‐19	2015	Lung	49	–	–
*Mycoplasma hyorhinis*	DE‐19‐08	2015	Lung	50	–	–
*Mycoplasma hyosynoviae*	3517/18J15	2015	Joint liquid	–	–	–
*Mycoplasma hyosynoviae*	421L19	2019	Lung	–	–	–
*Mycoplasma hyosynoviae*	4638L19	2019	Lung	–	–	–

^a^ Serovars determined by multiplex PCR (Howell et al., 2015) and high‐resolution melting PCR assay (Scherrer et al., 2022).

^b^ Virulence determined by leader sequence PCR (Galofré‐Milà et al., 2017).

### 2.2 Development of multiplex qPCR

Primers and probes were designed based on the following genes: HPS_219690793 (Howell et al., 2015) for identification of *G*. *parasuis*, *vtaA* for detection of potentially virulent *G. parasuis* strains (Galofré‐Milà et al., 2017), and the 16S ribosomal RNA (rRNA) genes for detection of *M*. *hyosynoviae* and *M. hyorhinis*. The design was achieved using CLC Main Workbench software 7.5.1 (Qiagen) from alignments of available sequences for HPS_219690793, *vtaA*, and 16S rRNA genes retrieved from the NCBI databank. Primers for *G. parasuis* were designed on gene HPS_219690793 with an amplicon length of 94 base pairs (bp), whereas the developed reverse primer (Primer_glaesserella_R) partly corresponded to the reverse primer published by Howell et al. (2015). Primers for the virulence marker of *G. parasuis* were designed on *vtaA* resulting in an amplicon length of 105 bp, while the forward primer (Primer_vtaA_F) partly corresponded to the published forward primer AV1‐F (Galofré‐Milà et al., 2017). Primers for *M. hyorhinis* and *M. hyosynoviae* were aimed at conserved regions of 16S rRNA genes amplifying a 63 bp fragment for *M. hyorhinis* and a 133 bp amplicon for *M. hyosynoviae* (Table 3). Oligonucleotide primers were synthesized by Microsynth. For each of the four targets, a specific probe was designed with fluorescent labeling and the respective reporter dye as indicated in Table 3. The probe for *G. parasuis* (Thermo Fisher Scientific) included a minor groove binding molecule at the 3ʹ‐end, allowing for a short 17 bp‐probe thereby increasing specificity. Probes detecting *vtaA*, *M. hyorhinis*, and *M. hyosynoviae* comprised Black Hole Quenchers (Eurogentec S.A.). All primer and probe sequences were tested for specificity by BLAST searches. For monitoring potential qPCR inhibition, eGFP was used as an internal amplification control as previously described (Hoffmann et al., 2006).

**Table 3:** Target genes, corresponding primers/probes, and characteristics of the multiplex qPCR.

Species	Gene	Name	Probe /primer (5′ → 3′)	Concentration probe/primer	Channel	*r* ^2^ Value	Efficiency (%)
*Glaesserella parasuis*	HSP_219690793	Probe_glaesserella	6‐FAM‐CGTTCGGCATTGACTAA‐MGB	50 nM	Green	0.999	96%
	HSP_219690793	Primer_glaesserella_F	AGCTTCCATAAAAGGG	300 nM			
	HSP_219690793	Primer_glaesserella_R	GGAATATCAGACAGGAG	300 nM			
*Glaesserella parasuis* virulence marker	*vtaA*	Probe_vtaA	ATTO680‐ACAACHACCCAAGCCTGTTGA‐BHQ3	75 nM	Crimson	0.999	102%
	*vtaA*	Primer_vtaA_F	AGAGTTATTTGGAGTCA	500 nM			
	*vtaA*	Primer_vtaA_R	GCATACTTGAGCTCT	500 nM			
*Mycoplasma hyorhinis*	16S rDNA	Probe_hyorhinis	YakkimaYellow‐TACCTAACCTACCTTTAAGACTGGGA‐BHQ1	100 nM	Yellow	0.997	95%
	16S rDNA	Primer_hyorhinis_F	ATGGGTGAGTAACACG	300 nM			
	16S rDNA	Primer_hyorhinis_R	AGCTATTGTTTCCAATAGTTA	300 nM			
*Mycoplasma hyosynoviae*	16S rDNA	Probe_hyosynoviae	Texas Red‐ATTCCGCTTACCTCTATCCAACTCT‐BHQ2	50 nM	Orange	0.998	100%
	16S rDNA	Primer_hyosynoviae_F	GTAGGCTGTTTATTAAGTCTG	300 nM			
	16S rDNA	Primer_hyosynoviae_R	CTTCCATATATCTACGCATTTC	300 nM			

Abbreviations: BHQ, Black Hole Quencher; MGB, minor groove binder; qPCR, quantitative PCR; rDNA, ribosomal DNA.

Multiplex qPCR experiments were executed using a Rotor‐Gene Q (Qiagen) and analyzed with the help of Rotor‐Gene Q Software 2.3.1 (Qiagen). The setup of the Rotor‐Gene instrument included an auto‐gain optimization step for each channel before starting with the first fluorescence acquisition at the beginning of the qPCR. The total reaction volume of the multiplex qPCR was 15 µL and consisted of 1 µL of genomic DNA (20 ng/µL), 1X QuantiNova Multiplex PCR Kit (Qiagen), 300 nM of primers targeting *G. parasuis*, *M. hyorhinis*, *M. hyosynoviae*, 500 nM of primers targeting *vtaA*, 200 nM of primers targeting eGFP, 50 nM of each probe detecting *G. parasuis* and *M. hyosynoviae*, respectively, 75 nM of *vtaA‐*probe, 100 nM of *M. hyorhinis‐*probe, 25 nM of eGFP*‐*probe, 1 µL (5 fg) eGFP plasmid DNA and ultrapure distilled water to complete the total reaction volume. The thermocycling conditions of the PCR were as follows: initial denaturation at 95°C for 2 min, 40 cycles with denaturation at 95°C for 5 s, and annealing/extension at 62.5°C for 30 s. The three reference strains *G. parasuis* serovar 1, *M. hyorhinis* ATCC 17981^T^, and *M. hyosynoviae* NCTC 10167^T^ were included in each qPCR run as positive controls. To exclude contaminations in the reaction mix, DNase‐ and RNase‐free water was tested as a negative control in each experiment. The multiplex qPCR was optimized using different concentrations of primer, probes, and annealing temperatures considering PCR efficiencies. Samples with a threshold cycle (*C*_t_) value of <35 were considered positive.

### 2.3 Pilot study

In a pilot study, different organs and swabs originating from 42 pigs from 31 farms were tested using the novel multiplex qPCR. Obtained samples (suspicious tissue from brain, joint, peritoneum, pleura, and nasal swabs) were first enriched in liquid tryptic soy broth at 37°C for 24 h. Afterward, sample disruption was achieved using Pathogen Lysis Tubes S (Qiagen) and the TissueLyser (Qiagen), according to the manufacturer's instructions. Subsequently, DNA was extracted using IndiSpin Pathogen Kit (Indical Bioscience) following the instructions in the kit handbook.

In parallel, obtained clinical samples were grown on Chocolate agar with Vitoyx and Columbia Blood agar (Thermo Fisher Diagnostics AG) at 37°C in 5% CO_2_ for up to 48 h and inspected for growth. To potentially cultivate *Mycoplasma* spp., swabs taken from infected tissue sites were inoculated in mycoplasma liquid media (Mycoplasma Experience Ltd.).

All directly extracted DNA samples were tested with the novel multiplex qPCR and compared to the results of culturing. Additionally, an HRM assay for serotyping *G. parasuis*‐positive samples (Scherrer et al., 2022) and a qPCR detecting *M. hyorhinis* and *M. hyosynoviae* (Gomes Neto et al., 2015) have been performed (Table A1).

### 2.4 Specificity

The specificity of the multiplex qPCR was determined by testing an exclusivity panel consisting of 23 pathogenic bacteria comprising 12 additional species not including *G. parasuis*, *M. hyorhinis*, or *M. hyosynoviae*. The following strains comprising three groups were tested: Bacteria involved in causing lesions similar to those caused in Glässer's disease, namely, *Streptococcus suis* (*n* = 5), *Erysipelothrix rhusiopathiae* (*n* = 1), *Escherichia coli* (*n* = 2), *Mycoplasma hyopneumoniae* (*n* = 1); nasal commensal microorganisms similar to *G. parasuis*: *Moraxella* spp. (*n* = 1) and *Neisseria animaloris* (*n* = 1); Gram‐negative coccobacilli, namely, *Actinobacillus suis* (*n* = 1), *Actinobacillus pleuropneumoniae* (*n* = 4), *Actinobacillus minor* (*n* = 1), *Pasteurella multocida* (*n* = 3), and *Bordetella bronchiseptica* (*n* = 2); and Gram‐positive rod *Listeria monocytogenes* (*n* = 1). Each strain was tested with a concentration of 20 ng/µL.

### 2.5 Analytical sensitivity

To determine the analytical sensitivities of the multiplex qPCR, the three reference strains *G. parasuis* serovar 1, *M. hyorhinis* ATCC 17981^T^, and *M. hyosynoviae* NCTC 10167^T^ were examined. With an estimated average genome size of 1.8 Mbp for *G. parasuis* (Brockmeier et al., 2014), 0.843 Mbp for *M. hyorhinis* (Cibulski et al., 2016; Käbisch et al., 2021; Trueeb et al., 2019), and 0.864 Mbp for *M. hyosynoviae* (GenBank accession number: CP008748.1), the following approximate DNA quantities corresponded to 1 genome equivalent (GE): 2 fg for *G. parasuis*, 0.95 fg for *M. hyorhinis*, and 0.93 fg for *M. hyosynoviae*. To obtain an accurate limit of detection (LoD) for each target and to identify a reasonable cut‐off *C*_t_ value, 20 replicates of each reference strain were analyzed with concentrations close to the LoD (*G. parasuis*: 1 GE, 10 GE, 100 GE, 200 GE, 500 GE, 2000 GE; *vtaA*: 1 GE, 100 GE, 200 GE, 1000 GE, 2000 GE, 5000 GE; *M. hyorhinis*: 1 GE, 20 GE, 100 GE, 200 GE, 300 GE, 1000 GE; *M. hyosynoviae*: 1 GE, 5 GE, 10 GE, 20 GE, 50 GE, 100 GE). The LoD at a confidence interval of 95% was defined as the concentration of DNA, where at least 95% positive replicates can be detected. The LoD was computed using the program GenEx version 7 (MultID Analyses AB) by plotting the fraction of positive samples against the concentration of the DNA template, and was illustrated at a logarithmic scale.

To examine the repeatability of the multiplex qPCR assay, the coefficients of intra‐ and inter‐assay variability were determined. For this purpose, reference strains *G. parasuis* serovar 1, *M. hyorhinis* ATCC 17981^T^, and *M. hyosynoviae* NCTC 10167^T^ were analyzed by using tenfold dilution series in a range between 100 and 10^7^ GE. DNA samples for each reference strain were measured in triplicates in three separate experiments.

### 2.6 Efficiency

To calculate efficiencies of the multiplex qPCR for each target, *C*_t_ values of dilution series measured in triplicates were plotted against input DNA concentrations, resulting in a standard curve for reference strains of *G. parasuis* serovar 1 and its virulence marker *vtaA*, *M. hyorhinis* ATCC 17981^T^, and *M. hyosynoviae* NCTC 10167^T^. The slope (*S*) of the standard curve, obtained by dilution series in the linear range between 10 and 10^7^ GE, was used to calculate PCR efficiency (*E*) as expressed in the following equation: *E* = (10^1/−S^ − 1) × 100.

### 2.7 Multiplex qPCR evaluation using field isolates

For further evaluating the multiplex qPCR, 20 ng of genomic DNA extracted from 21 *G. parasuis*, 26 *M. hyorhinis*, and 3 *M. hyosynoviae* field isolates were tested.

## 3 RESULTS

### 3.1 Multiplex qPCR amplification plots

**Figure 1:**
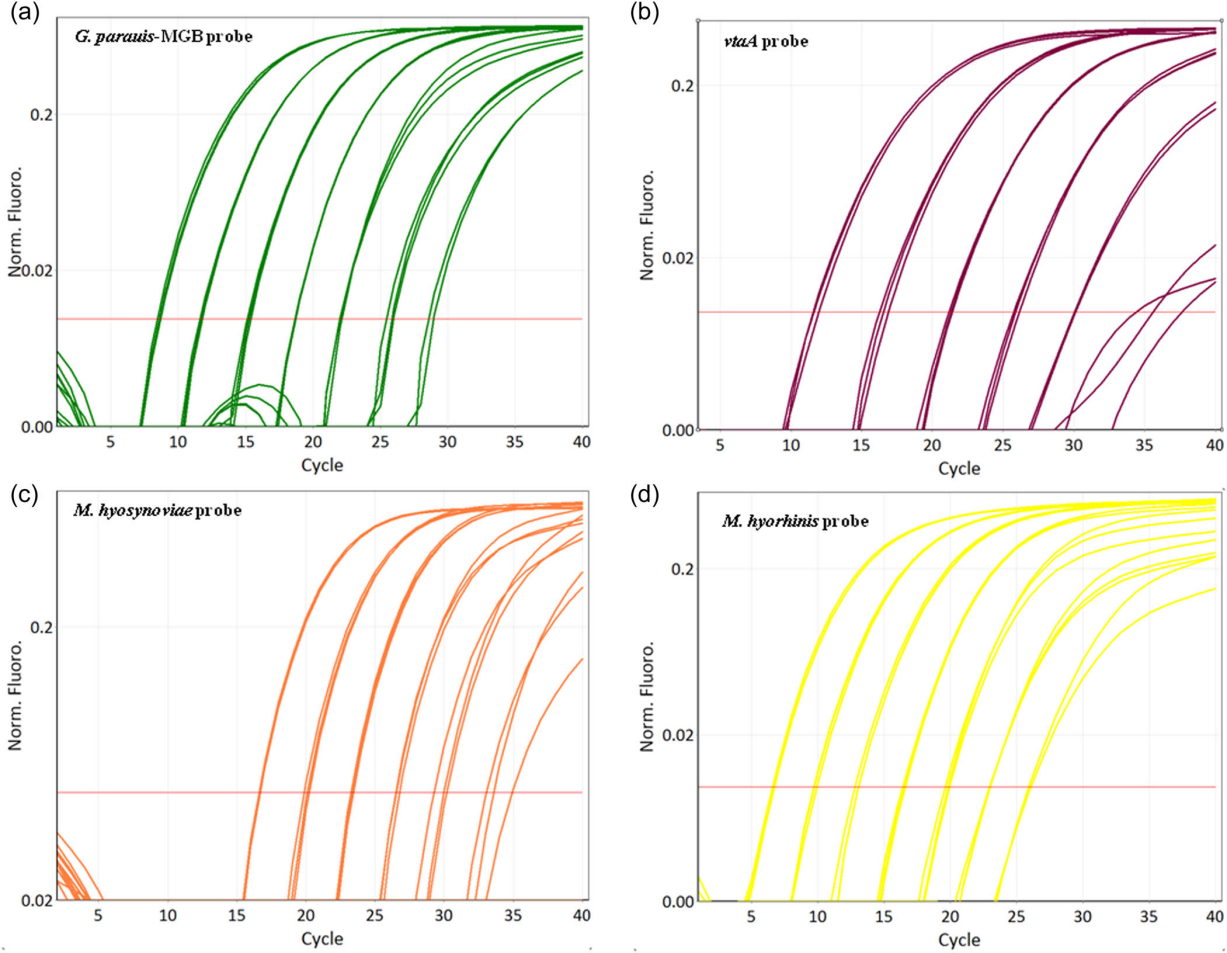
Performance of the multiplex qPCR. Dilution series (10–10^7^ genome equivalents) of *Glaesserella parasuis* serovar 1, *Mycoplasma hyorhinis* ATCC 17981^T^, and *Mycoplasma hyosynoviae* NCTC 10167^T^. Four channels are represented separately: (a) Channel green: probe 5ʹ‐FAM—MGB‐3ʹ detecting *Glaesserella parasuis*. (b) Channel crimson: probe 5ʹ‐ATTO680—BHQ3‐3ʹ detecting virulence marker *vtaA*. (c) Channel orange: probe 5ʹ‐TexasRed—BHQ2‐3ʹ detecting *M. hyosynoviae*. (d) Channel yellow: probe 5ʹ‐YakkimaYellow—BHQ1‐3ʹ detecting *M. hyorhinis*. BHQ, Black Hole Quenchers; MGB, minor groove binding; qPCR, quantitative polymerase chain reaction.

Amplification plots of the multiplex qPCR using different detection channels are shown in Figure 1. *G. parasuis, vtaA, M. hyosynoviae*, and *M. hyorhinis* can be recognized by channel green, crimson, orange, and yellow, respectively. The fifth channel red measures the internal control eGPF, which acts as an exogenous internal control monitoring the PCR for correct performance. For all samples measured, eGPF could be detected accurately.

### 3.2 Pilot study

A great variety of bacterial species was observed (most frequently in co‐culture with *S. suis*) and not in every case clinical signs could be unambiguously attributed to either *G. parasuis*, *M. hyorhinis*, or *M. hyosynoviae* as a causative pathogen. Additionally, up to three different *G. parasuis* serovars of both virulent and non‐virulent kinds could be detected in five animals. Due to the fastidious cultivation of the strains, in many cases, the successful isolation of involved strains was hampered. From the obtained 25 *G. parasuis*‐positive samples in the novel multiplex qPCR, six samples were identified to be non‐virulent (Table A1). The HRM assay detected 14 non‐virulent samples, seven virulent ones, and four cases, where brain and nasal samples of the same animal could be assigned as virulent and non‐virulent, respectively (Table A1). Most frequently, a mixture of several *G. parasuis* serovars could be detected. Amongst all samples, more efficient amplification of virulent *G. parasuis* could be observed when testing the novel qPCR, in contrast to the HRM assay using different primer pairs, which preferably seemed to amplify the fraction of non‐virulent *G. parasuis*. Overall, six isolates of *G. parasuis* could be generated from the pilot study (Table A1). In the case of *M. hyorhinis* and *M. hyosynoviae*, no isolate could be obtained due to a highly diverse accompanying bacterial flora. Comparing the results of the novel multiplex qPCR with a published qPCR detecting *M. hyorhinis* and *M. hyosynoviae*, the multiplex qPCR was able to identify *M. hyorhinis* in eight cases versus six cases when performing the published qPCR. *M. hyosynoviae* was found three times in both PCR assays, once each in a sample obtained from the pleura, joint, and peritoneum (Table A1).

### 3.3 Specificity

The tested exclusivity panel of 21 pathogenic bacteria resulted in negative results for all examined strains, thereby correlating with the expected in silico results. Hence, the new multiplex qPCR assay had a specificity of 100%.

### 3.4 Analytical sensitivity

**Figure 2:**
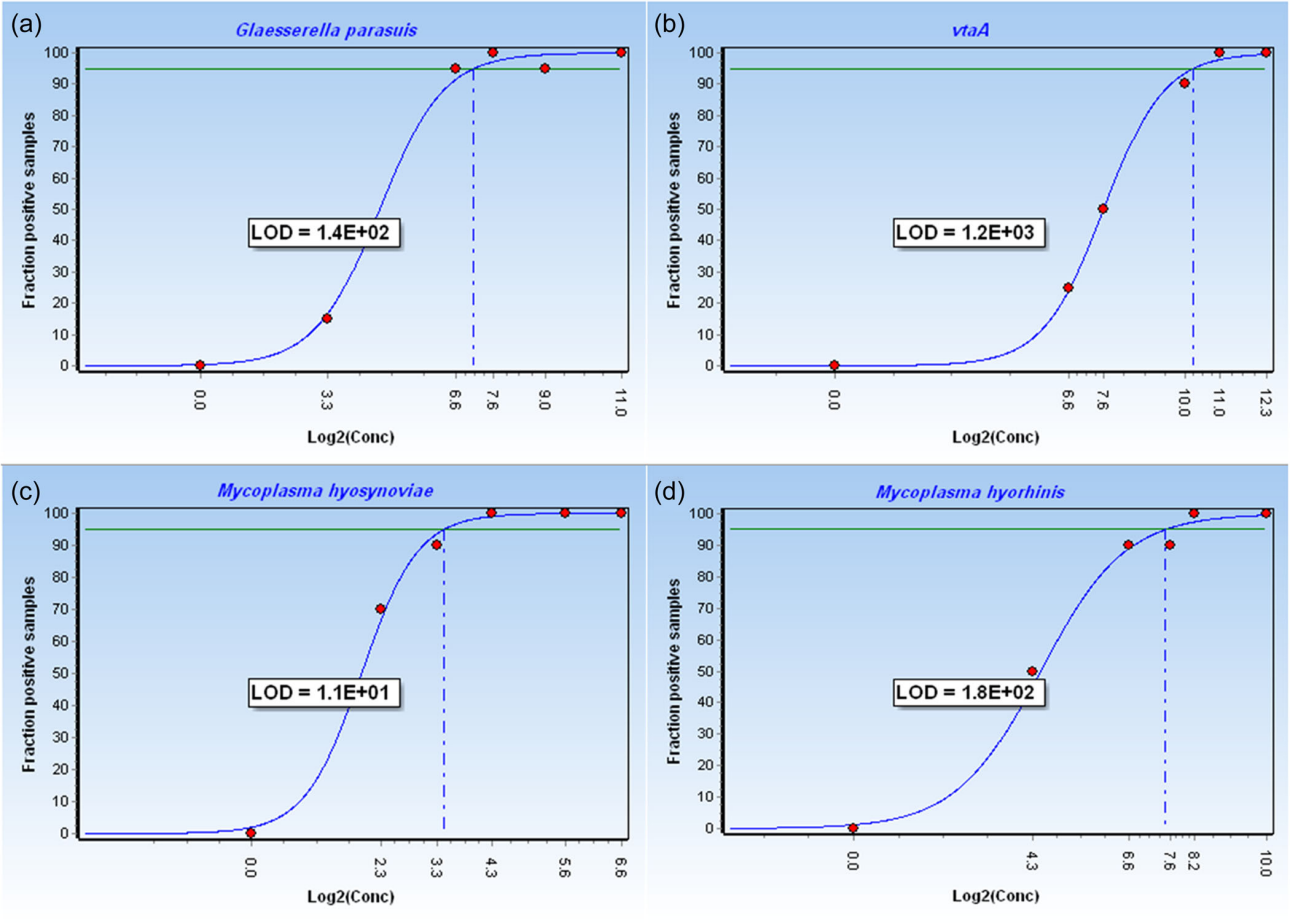
LoD for the four targets. Graphs illustrate the fraction of replicate samples with positive reads of dilution series at different concentrations in log scale. LoD is calculated at the relevant confidence level of 95% (green line). Data analysis was performed with GenEx version 7. The cut‐off threshold cycle value was 35. LoD is represented in GE for each target: (a) *Glaesserella parasuis*: 140 GE, (b) *vtaA*: 1200 GE, (c) *Mycoplasma hyosynoviae*: 11 GE, and (d) *Mycoplasma hyorhinis*: 180 GE. GE, genome equivalents; LoD, limit of detection.

The dynamic range of the standard curves was between 100 and 10^7^ GE for all reference strains. Within the relevant confidence level of 95%, the following LoD was reached for each target: 140 GE for *G. parasuis* corresponding to 200 fg–2 pg of DNA, 1200 GE for *vtaA* corresponding to 2–20 pg of DNA, 180 GE for *M. hyorhinis* corresponding to 100 fg–1 pg of DNA, and 11 GE for *M. hyosynoviae* corresponding to 10–100 fg of DNA, respectively (Figure 2). The cut‐off *C*_t_ value, which allows the correct interpretation of qPCR signals, was found to be *C*_t_ 35. The results of the variability assays revealed a coefficient of variation (CV) of CV% < 5% for the inter‐assay variability and CV% < 4% for the intra‐assay variability demonstrating the multiplex qPCR to be a reliable and highly reproducible assay (Table A2).

### 3.5 Efficiency

**Figure A1:**
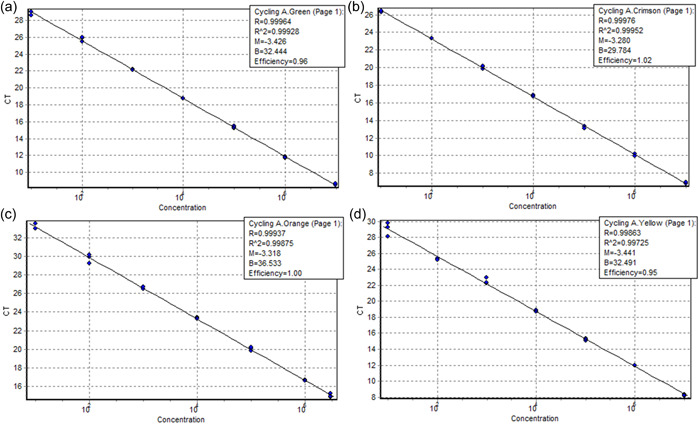
Standard curves of dilution series acquired by the multiplex qPCR in the range of 10–10^7^ genome equivalents. The following efficiencies were found: (a) 96% for *Glaesserella parasuis*, (b) 102% for *vtaA*, (c) 100% for *Mycoplasma hyosynoviae*, and (d) 95% for *Mycoplasma hyorhinis*. qPCR, quantitative PCR.

In the linear range of the tested dilution series, qPCR reactions had a high efficiency for each target species. qPCR efficiencies of 96%, 102%, 95%, and 100% were obtained for *G. parasuis, vtaA*, *M. hyorhinis*, and *M. hyosynoviae*, respectively. The correlation coefficient values of greater than 0.997 represented a good robustness and reproducibility of the qPCR assay (Figure A1).

### 3.6 Test evaluation with field isolates

DNA extracted from 21 *G. parasuis*, 26 *M. hyorhinis*, and 3 *M. hyosynoviae* field isolates were used for further evaluation of the established multiplex qPCR method. All field isolates could be identified unambiguously given a positive amplification curve measured in the respective color channel (Table A3).

## 4 DISCUSSION

Currently, diagnostic PCR assays using a one‐tube reaction system for the parallel detection of pathogens associated with polyserositis, polyarthritis, meningitis, pneumonia, or septicemia are missing. Moreover, the culture‐based detection of *Mycoplasma* is time‐consuming and tedious due to its slow growth, the requirement of complex media, and the frequent overgrowth by other bacterial species. Most diagnostic PCR tests are based on an approach to identify one pathogen per assay, such as qPCR assays detecting *M. hyorhinis* (Tocqueville et al., 2014) or *G. parasuis* (Cui et al., 2021; Turni et al., 2010). Furthermore, *M. hyosynoviae* and *M. hyorhinis* can be detected in one assay; though, the qPCR is performed in two separate reactions since different annealing temperatures are used (Gomes Neto et al., 2015). Recently, a TaqMan PCR assay was developed targeting multiple viral and bacterial porcine respiratory pathogens detecting eight viruses, eight bacteria, and one toxin in one approach, however, seventeen different reactions were performed in parallel (Sunaga et al., 2020). A cost‐effective and simple alternative to qPCR is a real‐time PCR assay coupled with high‐resolution melting, which has been successfully applied to discriminate between *Mycoplasma* species isolated from porcine and bovine respiratory disease in South Australia (Ahani Azari et al., 2020).

In a study analyzing a global set of *G. parasuis* isolates by LS‐PCR, the use of *vtaA* as a virulence predictor was shown to reliably detect most systemic isolates as virulent, and nasal isolates as nonvirulent, respectively (Macedo et al., 2021). However, in some cases, a small number of nondisease‐associated serovars, which were found to be non‐virulent by LS‐PCR, were recovered from systemic sites (Macedo et al., 2021). Co‐infections or different environmental factors such as stress or nonoptimal hygienic conditions might contribute to the invasion of less virulent strains into systemic sites (Galofré‐Milà et al., 2017; Turni et al., 2018). Overall, the high diversity of *G. parasuis*, when looking at serotyping and genotyping data of field isolates (Oliveira et al., 2003), and the multitude of strains with up to eight different strains as encountered on a farm in Australia (Turni & Blackall, 2010), reflect a great challenge for proper interpretation of the virulence of a strain.

In a pilot study, the application of clinical samples using the novel multiplex qPCR assay was tested by analyzing different clinical specimens from 42 diseased pigs. However, not in all cases a clear assignment of the examined isolates as a primary pathogen could be derived. Due to difficulties in obtaining pure cultures and isolating involved strains, we cannot prove the presence of the respective strains, thereby, impeding a coherent statement. Further studies will be needed to accurately correlate the virulence of *G. parasuis* to the corresponding phenotype and to determine the distribution of different *G. parasuis* serovars. Thus, the herein‐developed multiplex PCR lays a cornerstone for a planned future project testing porcine clinical samples in a comprehensive study to investigate the true prevalence of *G. parasuis*, *M. hyorhinis*, and *M. hyosynoviae* in pigs.

The novel qPCR has the potential to become a useful diagnostic tool to reliably identify and differentiate between *M. hyorhinis*, *M. hyosynoviae*, and pathogenic variants of *G. parasuis*. Nevertheless, the PCR mixture containing five differently labeled probes and ten oligonucleotide primers is rather complex, which can lead to some level of background signal interference. Thus, a careful setting of the threshold above the noise level is required when analyzing the results.

## 5 CONCLUSION

In conclusion, a sensitive and specific multiplex qPCR assay was developed for the identification of *G. parasuis* and its virulence factor *vtaA* differentiating between virulent and non‐virulent isolates, as well as *M*. *hyorhinis* and *M. hyosynoviae* revealing two important pig‐associated *Mycoplasma* species. The new multiplex qPCR is a simple one‐tube approach detecting three different swine pathogens in parallel, therefore allowing an efficient throughput of samples.

## AUTHOR CONTRIBUTIONS

**Simone Scherrer**: Conceptualization (equal); formal analysis (equal); investigation (equal); writing—original draft (lead). **Sarah Schmitt**: Conceptualization (equal); formal analysis (equal). **Fenja Rademacher**: Investigation (equal). **Peter Kuhnert**: Resources (lead); writing—review and editing (equal). **Giovanni Ghielmetti**: Conceptualization (equal). **Sophie Peterhans**: Conceptualization (equal); formal analysis (equal); investigation (equal). **Roger Stephan**: Conceptualization (equal); formal analysis (equal); writing—review and editing (equal).

## CONFLICT OF INTEREST STATEMENT

The authors declare no conflict of interest.

## ETHICS STATEMENT

The authors have nothing to report.

## Supporting information

Supporting information.

Supporting information.

Supporting information.

## Data Availability

All data relevant to the study are included in the article and its supporting information.
